# Drug resistance mutations and heteroresistance detected using the GenoType MTBDR*plus *assay and their implication for treatment outcomes in patients from Mumbai, India

**DOI:** 10.1186/1471-2334-12-9

**Published:** 2012-01-20

**Authors:** Monica Prem Tolani, Desiree Therese Blossom D'souza, Nerges Furdoon Mistry

**Affiliations:** 1 The Foundation for Medical Research., 84A, R. G. Thadani Marg, Worli, Mumbai 400 018, India

## Abstract

**Background:**

Only 5% of the estimated global multidrug resistant TB (MDRTB) load is currently detected. Endemic Mumbai with increasing MDR would benefit from the introduction of molecular methods to detect resistance.

**Methods:**

The GenoType MTBDR*plus *assay was used to determine mutations associated with isoniazid and rifampicin resistance and their correlation with treatment outcomes. It was performed on a convenience sample comprising 88 onset and 67 fifth month isolates for which phenotypic drug susceptibility testing (DST) was determined by the Buddemeyer technique for an earlier study. Simultaneous presence of wild type and mutant bands was referred to as "mixed patterns" (heteroresistance).

**Results:**

Phenotypically 41 isolates were sensitive; 11 isoniazid, 2 rifampicin, 2 pyrazinamide and 5 ethambutol monoresistant; 16 polyresistant and 78 MDR. The agreement between both methods was excellent (kappa = 0.72-0.92). Of 22 rifampicin resistant onset isolates, the predominant *rpoB *mutations were the singular lack of WT8 (n = 8) and mixed D516V patterns (n = 9). Of the 64 rifampicin resistant fifth month isolates, the most frequent mutations were in WT8 (n = 31) with a further 9 showing the S531L mutation. Mixed patterns were seen in 22 (34%) isolates, most frequently for the D516V mutation (n = 21). Of the 22 onset and 35 fifth month *katG *mutants, 13 and 12 respectively showed the S315T1 mutation with loss of the WT. Mixed patterns involving both S315T1 and S315T2 were seen in 9 and 23 isolates respectively. Seventeen of 23 and 23/35 *inhA *mutant onset and fifth month isolates showed mixed A16G profiles. Additionally, 10 fifth month isolates lacked WT2. Five onset and 6 fifth month isolates had both *katG *and *inhA *mutations. An association was noted between only *katG *but not only *inhA *resistance and poor outcome (*p *= 0.037); and additional resistance to ethambutol (*p *= 0.0033). More fifth month than onset isolates had mixed profiles for at least 1 gene (*p *= 0.000001).

**Conclusions:**

The use of the assay to rapidly diagnose MDR could guide simultaneous first- and second-line DST, and reduce the delay in administering appropriate regimens. Furthermore, detection of heteroresistance could prevent inaccurate "cured" treatment outcomes documented through smear microscopy and permit more sensitive detection of neonascent resistance.

## Background

Tuberculosis (TB), a leading cause of death globally, with increasing rates of drug resistance is of concern. Timely diagnosis and treatment are the key elements of the effort to combat TB and reduce transmission by rendering infectious cases non-infectious.

Only 5% of the World Health Organization (WHO) estimated global multidrug resistant TB (MDRTB) case load of 440,000 is currently detected [1,2]. Detection by conventional drug susceptibility testing (DST) requires considerable resources of infrastructure and trained personnel. The WHO recommends the use of MGIT960 and line probe assays (LPAs) towards quicker MDR detection [3] since phenotypic DST takes 4 to 6 weeks from the receipt of clinical samples.

Commercial and in-house systems for the rapid detection of rifampicin (RIF) resistant *Mycobacterium tuberculosis *(*M. tb*) take 5-8 hrs from the time of sample collection [4,5]. The GenoType MTBDR*plus *is superior in that it detects mutations associated with both rifampicin and isoniazid resistance unlike the INNO-LiPA Rif.TB (Innogenetics, Belgium) which detects mutations only to the former. Unlike RIF resistance, in which 95% of isolates have mutations within an 81-bp region of the *rpoB *gene encoding the RNA polymerase β subunit [6], isoniazid (INH) resistance has been associated with mutations in several genes [7,8]. Furthermore, since the technique is polymerase chain reaction (PCR) based, it allows detection of low levels of resistant bacteria amidst a predominantly susceptible population, providing a more accurate representation of the susceptibility of the infecting bacteria [9,10]. Different mutations lead to varying degrees of resistance and influence bacterial ability to multiply [11]. Studies have reported the potential use of sophisticated techniques such as sequencing to detect drug resistance mutations which can serve as epidemiological markers, since the relative frequency of alleles associated with resistance varies geographically [12-14]. The application of the GenoType MTBDR*plus *assay has been reported in other high burden settings such as Russia, South Africa and China, but has not yet been reported from our setting [10,15,16].

Despite guidelines that advocate DST for patients failing any treatment regimen [17], it is only performed in 0.5% of notified previously treated TB cases [1]. The endemic setting of Mumbai with reports of increasing levels of MDRTB [18-21] and a high case load would benefit from the introduction of molecular methods to detect resistance, overcoming the drawbacks of culture methods.

This study was therefore undertaken to evaluate the MTBDR*plus *for detection of MDR, defined as resistance to at least INH and RIF, in pulmonary TB (PTB) patients in Mumbai. The dual objectives thus encompassed determination of the nature and frequency of mutations associated with resistance and correlations, if any, between the type of mutation and treatment outcome of the patient. Additionally, the assay enabled the detection of heteroresistance to both INH and RIF.

## Methods

### Location and patient selection

This study was carried out on isolates from sputum samples collected from April 2004 to September 2007, for an epidemiological project on MDRTB transmission in Mumbai [18]. Samples were collected at onset and fifth month of treatment from patients registered with the DOTS (Directly Observed Therapy Short Course) Centers of the RNTCP (Revised National Tuberculosis Control Programme) in 4 centrally located wards, characterized by a high sputum positive case load and covering a population of 3 million. Patients were sputum smear positive, within the 15-70 yr age group, and commenced on 2 months of isoniazid, rifampicin, pyrazinamide and ethambutol followed by 4 months of isoniazid and rifampicin, each phase thrice weekly. To minimize bias towards MDR, patients with no history of TB or antitubercular therapy, and those with no interruption of treatment or interruption for < 2 weeks, were included after informed consent. Clearance for the parent study was obtained from the Foundation for Medical Research (FMR) Institutional Ethics Committee (20.07.2001/01).

A total of 681 onset and 345 fifth month samples were collected for the parent study. A convenience subset comprising of 88 onset and 67 fifth month samples covering different susceptibility profiles was used for this analysis.

### Drug susceptibility testing

Early morning samples were processed by the modified Petroff's method [22], stained by Ziehl-Neelsen Carbol Fuchsin, microscopically examined, and cultured on Lowenstein-Jensen (LJ) slopes (Himedia, India) and in Dubos broth (Himedia, India). Biochemical tests for niacin and catalase production were performed to confirm the identity of *M. tb*. DST was performed by the radiorespirometric Buddemeyer technique (a modification of the Bactec 460 technique) as described earlier [18,23,24] for the following drugs (μg/ml): Isoniazid (0.1), Rifampicin (2), Pyrazinamide (PZA-100) and Ethambutol (ETB-2.5).

MDR was defined as resistance to at least INH and RIF. Other cases were categorized as: sensitive - absence of resistance to any drug, monoresistance - resistance to only 1 drug and polyresistance - resistance to two or three drugs excluding the INH-RIF combination.

### External quality control of phenotypic DST

Ten percent of the isolates from the parent study were sent single blinded to the Swedish Institute for Infectious Disease Control, Stockholm, a WHO/International Union Against Tuberculosis and Lung Disease (IUATLD) supranational reference laboratory for external quality assurance by the Bactec 460 method. Kappa scores showed excellent agreement (range 0.76-0.77), for INH and RIF [18].

### The GenoType MTBDR*plus *assay (Hain Lifesciences, Germany)

DNA for the assay was extracted from bacilli grown on LJ media by the cetyl trimethyl ammonium bromide (CTAB) and chloroform isoamyl alcohol method [25]. The assay was performed according to the manufacturer's instructions. The test is based on DNA strip technology comprising multiplex PCR amplification and reverse hybridization.

#### Interpretation of results

Each strip consists of 27 reaction zones (bands), including 6 controls (conjugate, amplification, *M. tb *complex, *rpoB*, *katG*, and *inhA *controls), 8 *rpoB *wild-type [WT1-WT8 (506-509, 510-513, 513-517, 516-519, 518-522, 521-525, 526-529 and 530-533)] and 4 mutants [MUT1, 2A, 2B and 3 (D516V, H526Y, H526D and S531L)], 1 *katG *WT (315) and 2 mutants [MUT1 and 2 (S315T1 and S315T2)] and 2 *inhA *WT [WT1 and 2 (-15/-16 and -8)] and 4 mutants [MUT1, 2, 3A and 3B (C15T, A16G, T8C and T8A)].

In general for the 3 loci, a pattern comprising only WT bands was interpreted as sensitive. Resistance was interpreted as: i) absence of 1/more WT bands ii) presence of mutant bands with or iii) without the simultaneous absence of the complementary WT. The simultaneous presence of WT and corresponding mutant bands was referred to as a mixed pattern.

### Data analysis

Patterns of RIF and INH resistance in MDRTB and non MDRTB isolates were analysed. The ability of RIF resistance alone to predict MDR was also investigated. Results obtained through phenotypic DST and the GenoType MTBDR*plus *were compared for RIF, INH and MDR and kappa scores were generated for concordance. The interpretations were: < 0.45 = Poor, 0.45 - 0.70 = Fair and > 0.70 = Excellent [26]. The Chi square test using EpiInfo 2002 was used to determine association between parameters and a *p*-value of ≤ 0.05 was considered significant.

## Results

### Phenotypic DST

Amongst the 88 onset isolates, 41 (47%) were sensitive, 20 (23%) monoresistant (11 INH, 2 RIF, 2 PZA and 5 ETB), 18 (20%) MDR, and the remainder 9 polyresistant (10%). Amongst the 67 fifth month isolates, none were sensitive or monoresistant, 60 (90%) were MDR and 7 (10%) were polyresistant.

### Genotypic DST

Of the 88 onset isolates, 46 (52%) were sensitive, 20 (23%) only INH resistant, 2 (2%) only RIF resistant and 20 (23%) MDR. Of the 67 fifth month isolates 1 (1.5%), 2 (3%), 2 (3%) and 62 (92.5%) were sensitive, only INH resistant, only RIF resistant and MDR respectively.

### Comparison of the Buddemeyer and GenoType MTBDR*plus *assays

Excellent agreement was obtained between the 2 methods for the onset, fifth month and combined isolates for INH and RIF as represented in Table 1.

**Table 1:** Concordance between genotypic (MTBDR*plus *assay) and phenotypic (Buddemeyer assay) resistance

Sample	INH		RIF	
	
	% Concordance	kappa	% Concordance	kappa
**Onset (n = 88)**	86	0.72	94	0.84

**5th month (n = 67)**	97	0.74	99	0.85

**Cumulative (n = 155)**	91	0.80	96	0.92

A total of 73 (83%) onset and 64 (96%) fifth month isolates showed concordant DST profiles for INH and RIF (Table 2). A comparison of the genotypic versus phenotypic assays at onset revealed 7 isolates which were INH resistant and RIF sensitive by the GenoType MTBDR*plus *assay but INH and RIF sensitive by the Buddemeyer assay. Additionally, 2 isolates were INH and RIF sensitive by the GenoType MTBDR*plus *assay but INH resistant and RIF sensitive by the Buddemeyer assay (Table 2). Of 4 isolates identified MDR by the MTBDR*plus*, 3 were phenotypically only INH resistant, while 1 was only RIF resistant. Amongst the fifth month isolates, discordance was noted in 3 isolates identified as MDR by the MTBDR*plus*, wherein the Buddemeyer detected 1 to be only INH resistant and 2 to be only RIF resistant.

**Table 2:** Comparison of the MTBDR*plus *assay with conventional drug susceptibility testing at onset and 5th month

		GENOTYPIC DST			
**PHENOTYPIC DST**		**INH^s ^RIF^s^**	**INH^r ^RIF^s^**	**INH^s ^RIF^r^**	**INH^r ^RIF^r^**

*Onset (n = 88)*	INH^s ^RIF^s^	43	7	0	0
	
	INH^r ^RIF^s^	2	13	0	3
	
	INH^s ^RIF^r^	0	0	1	1
	
	INH^r ^RIF^r^	1	0	1	16

*5th month (n = 67)*	INH^s ^RIF^s^	1	0	0	0
	
	INH^r ^RIF^s^	0	2	0	1
	
	INH^s ^RIF^r^	0	0	1	2
	
	INH^r ^RIF^r^	0	0	1	59

### Mutations identified using the GenoType MTBDR*plus *assay

The frequency of mutations detected in the 3 genes is described in Table 3.

**Table 3:** Frequency distribution of mutations across *rpoB*, *katG *and *inhA *genes

Pattern	*rpoB*	*katG*	*inhA*	n (onset)	n (5th month)
**Only INH (n = 22)**	-	-	A16G mixed	6	1
	
	-	S315T1	-	3	1
	
	-	-	ΔWT2	2	0
	
	-	-	C15T	2	0
	
	-	S315T1, T2	-	2	0
	
	-	S315T1, T2 mixed	-	2	0
	
	-	S315T1, T2	A16G mixed	1	0
	
	-	S315T1 mixed	-	1	0
	
	-	S315T1, T2 mixed	A16G mixed	1	0

**Only RIF** **(n = 4)**	Δ WT8	-	-	1	1
	
	ΔWT6, 7, 8	-	-	0	1
	
	D516V mixed	-	-	1	0

**MDR** **(n = 82)**	ΔWT8	S315T1, T2 mixed	-	2	11
	
	D516V mixed	-	A16G mixed	4	5
	
	ΔWT8	-	ΔWT2	1	6
	
	ΔWT8	-	A16G mixed	3	4
	
	S531L	S315T1	-	1	4
	
	D516V mixed, ΔWT8	-	A16G mixed	0	4
	
	S531L	S315T1, T2 mixed	-	1	3
	
	D516V mixed, S531L	S315T1, S315T2	-	0	3
	
	ΔWT8	S315T2 mixed	-	1	1
	
	ΔWT7, 8	-	ΔWT2	0	2
	
	D516V mixed, ΔWT8	S315T1, T2 mixed	A16G mixed	0	2
	
	D516V mixed, ΔWT8	S315T1, T2 mixed	-	0	2
	
	D516V mixed	S315T1, S315T2	A16G mixed	1	1
	
	ΔWT8	S315T1, S315T2	-	0	1
	
	ΔWT4	S315T1, S315T2	-	1	0
	
	ΔWT8	-	C15T mixed	0	1
	
	ΔWT8	S315T1 mixed	-	0	1
	
	ΔWT7	-	ΔWT2	0	1
	
	ΔWT6, 7, 8	-	ΔWT2	0	1
	
	ΔWT1, 6, 8	-	A16G mixed	0	1
	
	H526Y	S315T1, S315T2	-	0	1
	
	S531L	S315T1, S315T2	C15T	1	0
	
	S531L	S315T1, S315T2	-	1	0
	
	D516V mixed, S531L	S315T1, T2 mixed	-	1	0
	
	D516V mixed, S531L	S315T1, S315T2	A16G mixed	1	0
	
	D516V mixed, S531L	-	C15T	0	1
	
	S531L	S315T1, S315T2	A16G mixed	0	1
	
	S531L	-	A16G mixed	0	1
	
	D516V mixed, S531L	S315T1, T2 mixed	A16G mixed	0	1
	
	D516V mixed, S531L	S315T2 mixed	-	0	1
	
	D516V mixed, S531L	-	A16G mixed	0	1
	
	D516V mixed	S315T1, S315T2	-	1	0
	
	S531L mixed	S315T1, T2 mixed	A16G mixed, T8A	0	1

#### RpoB

Twenty-two onset and 64 fifth month isolates showed resistance. For the onset isolates, the most frequent mutation was the absence of only WT8 (n = 8). A further 4 isolates showed the corresponding S531L mutation. Mixed patterns for D516V were seen in 9 isolates. Of the fifth month isolates, the most frequent mutations were in codons 530-533 (n = 31), wherein 26 showed the absence of WT8 alone; or in combination with absence of WT6, 7 (n = 2); WT7 (n = 2) or WT1, 6 (n = 1). A further 9 isolates showed the corresponding S531L mutation. Only 1 isolate showed a H526Y mutation. Mixed patterns were seen in 22/64 isolates (34%), the most frequent for the D516V mutation (n = 21). For those WT bands with a corresponding mutant band (WT 3, 4, 7 and 8), loss of WT only was seen in 9 onset and 40 fifth month isolates.

#### KatG

Resistance was detected in 22 onset and 35 fifth month isolates. Lack of the wild type with the presence of the S315T1 mutation were seen in 13 onset and 12 fifth month isolates, of which 9 and 7 respectively also showed the S315T2. Mixed patterns involving both mutations were seen in 9 and 23 isolates respectively. Amongst onset isolates, an association was noted between *katG *mutations and resistance to ethambutol (*χ*^2 ^= 8.63, *p *= 0.0033).

#### InhA

Of 23 isolates resistant at onset, 3 each showed either only absence of WT2 or the C15T mutation. Seventeen showed mixed profiles for A16G. Of 35 isolates resistant at fifth month, 10 lacked the WT2, while 23 had a mixed profile for A16G, of which 1 had a mixed profile even for T8A. Loss of WT only was seen in 3 onset and 10 fifth month isolates.

Analysed collectively, 40 onset and 64 fifth month isolates showed INH resistance. Of these, 17 onset (43%) and 29 fifth month (45%) isolates showed resistance only through *katG *while in both groups an equivalent proportion [45% (18 onset and 29 fifth month)] were resistant only through *inhA*. The remaining 5 onset and 6 fifth month isolates had mutations in both genes.

Overall, 46 onset and only 1 fifth month isolate were sensitive to INH and RIF. Of 18 onset and 4 fifth month isolates resistant to only INH, 2 onset isolates had dual mutations in *katG *and *inhA*, while the remainder had mutations in either 1. No fifth month isolates had such dual mutations. Two isolates each among the onset and fifth month isolates were resistant to only RIF (Table 3).

Of the 82 MDR isolates (20 onset and 62 fifth month), the predominant patterns involved absence of WT8 with either S315T1/2 or Δ-8 mutations (Table 3). No association was found between a particular mutation and a monoresistant or MDR profile.

An analysis of the profiles for all 3 genes revealed that a significant number of fifth month isolates had mixed profiles in comparison to onset isolates (*χ*^2 ^= 23.75, *p *= 0.000001). For *katG*, an association (*p *= 0.0012) was noted between a clean resistant profile (as against a mixed profile) and poor outcome (death or treatment failure). Similarly an association was noted between only *katG *resistance (as against only *inhA *resistance) and poor outcome (*χ*^2 ^= 4.37, *p *= 0.037). Cure was associated with A16G (*p *= 0.02) and Δ-8 (*p *= 0.015) in comparison to a C15T mutation in the *inhA*.

### Rifampicin resistance as an indicator of MDRTB

Of the 23 isolates showing RIF resistance at onset by the MTBDR*plus*, 2 were only RIF resistant, whilst the remainder were MDR. Of the fifth month isolates, 62/64 RIF resistant isolates were MDR.

## Discussion

To the best of our knowledge this is the first study from this setting to investigate genotypic profiles in the *rpoB*, *katG *and *inhA *regions associated with DR using the MTBDR*plus *assay. Techniques which detect MDR mutations in new cases at onset or during therapy would enable rapid identification of MDR and facilitate the modification of regimens with improvement to programme practices.

Overall, the concordance between the methods for INH and RIF ranged from 86-97% and 94-99% respectively. Fifteen discordant results were obtained for INH resistance, of which 5 were phenotypically resistant but genotypically sensitive, possibly because the mutations lie outside the regions covered by the probes. Ten discordant results were Buddemeyer sensitive but MTBDR*plus *resistant, wherein 5 were *inhA *mutants, known to be associated with low level resistance [27]. Of the remainder, 4 were onset isolates with *katG *mutations, of which 2 showed a mixed profile. This may explain the phenotypic sensitivity despite the *katG *mutations being associated with high level resistance [27]. The remainder 2 profiles showed the *katG *S315T1 mutation but were INH sensitive by the Buddemeyer. For one of these isolates, the interpretation of the drug susceptibility could have altered from sensitivity to resistance on extended incubation. For the second isolate, there was no indication of resistance and the discordance could reflect a technical anomaly in the phenotypic assay, or an "adaptation" by the strain which has prevented phenotypic expression of the *katG *mutation.

Of 5 isolates that were MTBDR*plus *RIF resistant but Buddemeyer sensitive, 4 lacked the WT8 band, including 3 which showed the MUT3 (S531L). Though associated with high level resistance, its detection at onset could imply a smaller proportion of resistant bacteria. Low level resistance which may remain undetected despite conventional DST has been previously reported [28,29].

Heteroresistance, reflecting the slow evolution of bacteria from a sensitive to resistant profile, is not uncommon in *M. tb *[30]. However our study is probably the first to report relatively high levels of heteroresistance in all 3 genes in an endemic setting. Two explanations are offered for this finding. Firstly, heteroresistance could have arisen due to transmission of both susceptible and resistant bacterial populations from drug resistant patients to previously untreated cases. An endemic setting like Mumbai would be prone to the prevalence of MDR strains, and hence new cases, even at onset, are likely to harbour resistant bacteria at proportions genotypically detectable but phenotypically undetectable, as suggested by the detection of mixed profiles in our onset isolates [31]. Studies on heteroresistance have shown that phenotypic DST results corresponded to the mutated, i.e. resistant, organism [31]. Secondly, the presence of exclusively sensitive bacteria at onset which gradually develop resistance during therapy, with incomplete elimination of the sensitive population by fifth month, would result in phenotypic resistance but with both forms remaining detectable genotypically. This possibility has been explained through mathematical modelling of the scenario in which MDR bacilli arise from a completely sensitive original infection [32]. The second scenario may explain the higher occurrence of mixed patterns among our fifth month isolates in comparison to the onset isolates. The probability of any bias towards detection of heteroresistance has been reduced by excluding patients with various other likely contributory factors such as prior treatment and defaulting. DNA fingerprinting techniques such as Mycobacterial Interspersed Repetitive Units Variable Number Tandem repeats (MIRU VNTR) would help gauge whether multiple infections have contributed to the heteroresistance detected.

The detection of heteroresistance seems to support our finding of an association between a clean resistant profile for the *katG *and a poor outcome, since the presence of exclusively resistant strains is more likely to result in non responsiveness to treatment. Identification of hetereoresistance can be used to probe outcomes of smear examination based "cure" since microscopy may not be sensitive enough to detect a small focus of bacteria which have evolved from sensitivity to resistance during therapy.

INH resistance can develop through mutations in the *inhA *open reading frame (ORF) (0-5%) or the promoter (8-20%). Between 40 to 95% of INH resistant isolates have mutations in *katG*, 75-90% of which are in codon 315, with 10-25% in other loci [33]. The frequency of the *katG *S315T substitution in *M. tb *strains varies globally in relation to the prevalence of TB: from 26-30% in regions with intermediate/low prevalence [34] upto 91% of strains in Russia [35]. Most reports reveal higher levels of *katG *mutations in comparison to the *inhA *mutations, viz. 73% and 22% [36], 46% and 27% [37], 64% and 42% [10] respectively. However we detected nearly equivalent levels of *katG *- 55% at onset and fifth month; and *inhA *- 58% onset and 55% fifth month in concurrence with Lacoma et al. [27]. We found dual mutations in *katG *and *inhA *in 12.5% and 9% of onset and fifth month isolates, comparable to the 3-13% reported elsewhere [2,15,38].

Mutations in *katG *315 may be favoured because they decrease INH activation without abolishing catalase-peroxidase activity, reflecting its low fitness cost [11,33,39-41]. The detection of equal proportions of *katG *and *inhA *mutations indicate that in our setting, *inhA *may also have a low fitness cost. This may be a consequence of the extended occurrence of MDR allowing for acquisition of compensatory mutations such as in the *ahpC *[42]. Despite their equivalent levels, *katG *but not *inhA *mutations, were associated with treatment failure.

Our data identified associations between *katG *315 mutations and ethambutol resistance as well as poor outcome. It has been reported that the *iniBAC *promoter is induced by cell wall biosynthesis inhibitors such as isoniazid and ethambutol [43]. Overexpression of the *iniA *gene confers a tolerance-like phenotype to INH and ETB. Furthermore, the *iniA *gene product is an essential component of an MDR-like pump [44]. Resistance to INH through *katG *mutations might influence response to other drugs, allowing development of resistance to ethambutol [45] and streptomycin [46], leading to poor treatment response.

Of the 86 RIF resistant isolates, 70 showed a mutation in the 530-533 region of the *rpoB*. However only 23 (27%) showed the specific S531L mutation as compared to other studies reporting 46-79% of strains with this mutation [10,27,36,37]. The low fitness cost of *rpoB *S531L [47] may account for its high frequency in these regions (i.e. South Africa, France, Spain) though its occurrence has also been reported to be as low as 30-31% in India and Hungary [12,48]. The lower proportion of the S531L compared to the D516V mutation indicates that the latter is also not associated with a fitness cost, at least in our setting.

Our study failed to find any association between a particular mutation and the occurrence of monoresistance or MDR. However, other studies have reported a significantly higher level of *katG *and S531L mutations in MDR isolates compared to INH or RIF monoresistant isolates respectively [10,38]. It is likely that this difference is due to the relatively low occurrence of S531L and the equivalent proportions of *katG *and *inhA *mutations in our cohort.

The value of RIF as a surrogate MDR marker has been documented [49] and further corroborated in our study. Despite their advantages, genotypic methods do not always identify phenotypically resistant strains [38], highlighting the limitations of molecular testing and need for supplementation with culture or additional probes. Additionally resistance can be inferred from the absence of a wild type signal alone, without confirmation by a mutant probe signal (in 47% of our isolates) and may be due to a mutation in a region not associated with resistance [29]. Such susceptible isolates would be called resistant leading to the unnecessary removal of RIF and/or INH from therapy. Moreover since a proportion of INH resistance, particularly in monoresistant isolates, could be due to resistance determinants other than *katG *S315T and *inhA *C15T, these isolates would also be indicated as susceptible. This highlights the need for the interpretation of genotypic data in conjunction with patient clinical status and the determination of mutations specific to certain geographical locales.

## Conclusions

Despite the limitations of the MTBDR*plus *assay, its rapidity and sensitivity still favour its implementation as an initial screen for MDRTB. The presence of resistance mutations and the occurrence of heteroresistance could not only guide the need for simultaneously initiating first- and second-line DST, but also significantly reduce the delay in administering an appropriate regimen to an MDR (or extensively drug resistant) TB case.

## Competing interests

No author has a financial or other conflict of interest related to this work. None of the authors have an association that poses any conflict of interest. The funders had no part in the decision to publish the manuscript.

## Authors' contributions

MPT and DTBD performed the assays, performed the analysis and drafted the manuscript. NFM provided inputs into data analysis and interpretation and editing of the manuscript. All authors read and approved the final manuscript.

## Pre-publication history

The pre-publication history for this paper can be accessed here:

http://www.biomedcentral.com/1471-2334/12/9/prepub
